# The mediating role of partner support in the relationship between reproductive health concerns and psychological distress among cancer survivors

**DOI:** 10.1038/s41598-026-53124-z

**Published:** 2026-05-25

**Authors:** Shaimaa Mohamed Amin, Sally Mohammed Farghaly Abdelaliem, Amany Anwar Saeed Alabdullah, Ibrahim Alasqah, Nagwa Ibrahim Elfeshawy, Heba Saied Ibrahim Ali, Mahmoud Abdelwahab Khedr, Eman Ahmed El-kholy, Mohamed Hussein Ramadan Atta, Ahmed Hashem El-Monshed, Ayman Mohamed El-Ashry

**Affiliations:** 1 Department of Community, Psychiatric, and Mental Health Nursing, College of Nursing, Qassim University, 51452 Buraydah, Saudi Arabia; 2 Community Health Nursing Department, Faculty of Nursing, Damanhour University, Elbehaira, Egypt; 3 School of Health, University of New England, 2351 Armidale, NSW Australia; 4 Department of Nursing, Faculty of Allied Health Sciences, Kuwait University, Kuwait City, Kuwait; 5 Department of Nursing Administration, Faculty of Nursing, Alexandria University, Alexandria, Egypt; 6 Department of Maternity and Pediatric Nursing, College of Nursing, Princess Nourah bint Abdulrahman University, P.O. Box 84428, 11671 Riyadh, Saudi Arabia; 7 Nursing Services Department, Qassim University Medical City, Buraydah , 52571 Saudi Arabia; 8 Women’s Health and Midwifery Nursing, Faculty of Nursing, Mansoura University, Mansoura, Egypt; 9 Obstetrics and Gynecology Nursing Department, Faculty of Nursing, Alexandria University, Alexandria, Egypt; 10 Psychiatric and Mental Health Nursing Department, Faculty of Nursing, Alexandria University, Alexandria, Egypt; 11 Maternal and Neonatal Health Nursing, Faculty of Nursing, Tanta University, Tanta, Egypt; 12 Department of Nursing, College of Health and Sport Sciences, University of Bahrain, Manama City, Bahrain; 13 Department of Psychiatric and Mental Health Nursing, Faculty of Nursing, Mansoura University, Mansoura , Egypt; 14 Maternal and Pediatric Nursing, College of Nursing, King Khalid University, Abha, Saudi Arabia; 15 Basic Nursing Department, Faculty of nursing, Isra university, Amman, Jordan; 16 Nursing Department, College of Applied Medical Sciences, Prince Sattam Bin Abdulaziz University, Wadi Addawasir City, Saudi Arabia

**Keywords:** Cancer, Mediating effect model, Male partner, Psychological distress, Reproductive health concerns, Mental health

## Abstract

Cancer survivorship often includes reproductive health concerns (e.g., fertility potential and treatment‐related health problems) that elevate psychological distress. Partner support is a key, modifiable resource linked to better adjustment, yet its pathway of influence is not fully understood. To test whether partner support mediates and/or moderates the association between reproductive health concerns and psychological distress among female cancer survivors. A cross-sectional study was conducted with 202 married female cancer survivors (ages 20–49) attending the oncology departments of Tanta University Hospital, Egypt. Validated measures included the Reproductive Concerns after Cancer Scale, Partner Support Scale, and Kessler Psychological Distress Scale. Higher reproductive concerns related to greater distress (r = 0.517, *p* < 0.01). Partner support correlated negatively with reproductive concerns (r =  − 0.446, *p* < 0.01) and distress (r =  − 0.471, *p* < 0.01). Adding partner support improved prediction of distress (Model 2, R^2^ = 0.345); partner support was a significant negative predictor (B =  − 0.364, *p* < 0.001). Mediation was supported: Reproductive Concerns after Cancer Scale → distress total effect B = 0.4212 (*p* < 0.001); direct effect B = 0.3124 (*p* < 0.001); indirect effect via partner support B = 0.1088, 95% CI [0.0617, 0.1613]. Moderation was not supported (interaction B≈0.000, *p* = 0.998). Conclusions: Reproductive concerns are associated with elevated psychological distress. Partner support partially mediates yet does not moderate this relationship, indicating a protective, indirect effect on distress. Implications for Practice: Integrate structured partner‐involved education and counselling into survivorship care to reduce distress linked to reproductive concerns (confirmed need). Screen routinely for reproductive concerns and perceived partner support and refer dyads to targeted psychosocial interventions. Training for nurses should prioritize communication about fertility/health concerns and techniques to engage partners effectively.

**Supplementary Information:**

The online version contains supplementary material available at 10.1038/s41598-026-53124-z.

## Introduction

Young adult cancer is a growing global concern, especially for women of childbearing age^1^. In 2022, almost 1.25 million adults aged 20–40 years were diagnosed with a new cancer^2,3^. This is disproportionately affected by women, who account for 63% of cases^2^. In particular, 15.25% of new cancer cases were gynecological cancers^4^. Despite these challenges, improvements in cancer care have improved survival, with more than 70% of cases diagnosed under the age of 45 surviving more than five years after cancer treatment^5,6^.

Cancer survivorship is a multidimensional concept that includes physical, psychological, and social concerns^7^. A key area of concern relates to reproductive health, especially for those diagnosed in their reproductive years^8^. Consistent with the World Health Organization (WHO) definition of reproductive health, these concerns are any physical, social, and psychological issues affecting the reproductive system^9^. These concerns among cancer survivors include distress related to fertility, sexual activity, hormone changes, relationships, body image, and the capacity to have or complete a family^9^.

Fertility problems may be a direct consequence of cancer or an indirect consequence of cancer treatments, including chemotherapy, radiotherapy, hormonal therapy, and surgery^10^. Reproductive concerns, however, are broader and may arise even in the absence of confirmed infertility, as survivors may worry about fertility potential, pregnancy safety, sexual health, hormonal changes, body image, and future family planning^11–13^. One recent study found that 35–38% of survivors are less likely to conceive than healthy controls, revealing the effects of these treatments on fertility^14,15^. In general, reproductive concerns are common among female cancer survivors (Overall, reproductive concerns are common among female cancer survivors, with reported prevalence ranging from 44 to 86%. Among these survivors, approximately 28% to 44% report moderate to severe reproductive concerns)^16^.

These concerns of reproductive function increase psychological distress^17,18^, which is characterized as an unpleasant emotional experience that includes psychological, social, and spiritual components that interfere with a survivor’s ability to cope with cancer symptoms and treatment^19^. It results in negative body image, lowered self-disclosure, and hindered psychological and social adaptation. Moreover, it has a profound effect on treatment decision-making, which leads to regret, lack of hope, and lower treatment adherence, resulting in lower quality of life^10,20^. This is largely due to the fear of cancer reappearing^21^. However, psychological distress of cancer does not affect only the individual, but also the couple’s relationship^22^.

In cancer survivorship, partner support has been shown to play an important role in adjustment to the disease and in maintaining relationship well-being^23–26^. In the present study, which focuses on married female cancer survivors, the intimate partner is considered a key source of emotional, practical, and decision-making support, especially in relation to reproductive concerns, fertility-related decisions, and psychological adjustment^27^.

In the case of female cancer survivors, the partner’s support is a mixed blessing. While according to the social support theory, positive social support from a partner is related to a higher quality of life and a positive psychological health assessment^28,29^, it can also be problematic support, defined as well-meaning social exchanges that are perceived as unhelpful or harmful^30^. A recent study on breast cancer survivors found that some forms of support, such as overprotection or unsolicited advice, may be perceived as unhelpful and can negatively affect survivors’ self-perception, autonomy, emotional adjustment, and relationship outcomes^31^. Lastly, the success of this support is contingent on the person’s level of optimism, emotional resilience, and realistic perceptions of his/her abilities^32^. Therefore, partner support is important to examine as a potential mediator and moderator of the relationship between reproductive concerns and psychological distress among married female cancer survivors.

Reproductive concerns may affect cancer survivors across sex and gender groups; however, the present study focuses specifically on married female cancer survivors of reproductive age and examines perceived support from their spouse or intimate partner. Women diagnosed during their reproductive years may experience cancer- and treatment-related reproductive issues, including reduced fertility potential, menstrual and hormonal changes, premature menopause, pregnancy-related concerns, and worries about having or completing a family^10–13,16^. These concerns are especially relevant in survivorship care because they may contribute to psychological distress and may influence reproductive decision-making within the marital relationship. Although the association between reproductive concerns and psychological distress among cancer survivors has been established^33^, the role of partner support in this relationship remains less clear. Therefore, this study tests whether partner support mediates and/or moderates the association between reproductive health concerns and psychological distress. As a mediator, partner support may help explain how reproductive concerns are associated with psychological distress through survivors perceived level of support. As a moderator, higher partner support may reduce the strength of the association between reproductive concerns and psychological distress. By examining both pathways, this study contributes to social support theory and provides evidence to guide partner-focused psychosocial support.

### Study hypotheses

#### H1

Female cancer survivors will report a high prevalence of reproductive health concerns and psychological distress.

#### H2

Partner support will significantly mediate the relationship between reproductive health concerns and psychological distress.

#### H3

Partner support will significantly moderate the relationship between reproductive concerns and psychological distress, such that higher partner support will weaken the association between reproductive concerns and psychological distress.

### Study design and setting

This study employed a cross-sectional descriptive research design, following the STROBE checklist for cross-sectional studies to ensure methodological rigor. Data collection was conducted at the Oncology Departments of Tanta University Hospital, which is affiliated with the Ministry of Higher Education in Egypt. These oncology departments are renowned centers for cancer care, education, and research. They play a critical role in delivering comprehensive medical services to cancer patients, including diagnosis, treatment, and ongoing care. The departments are equipped with state-of-the-art medical technology and staffed by a multidisciplinary team of oncologists, nurses, psychologists, and social workers, ensuring a well-rounded and holistic approach to cancer care.

### Sample size and study population

The sample size was calculated using G*Power software, version 3.1.9.7 (Heinrich Heine University Düsseldorf, Düsseldorf, Germany), available at: https://www.gpower.hhu.de/^34^. The analysis was performed with a 95% confidence level, a 5% alpha (significance) level, and a medium effect size of 0.15^35^. Given the complexity of the regression analysis, which included 15 predictors (covering Reproductive Concerns After Cancer, Psychological Distress, Partner Support, and Psychological Distress) and 12 demographic and clinical variables, the minimum required sample size was calculated to be 199 participants. To account for potential challenges related to the chosen data collection method and an anticipated 30% dropout rate^36^, we decided to recruit an additional 60 participants. This resulted in a target sample size of 259 participants. However, by the conclusion of the study, 202 participants successfully completed the survey.

This study focused on married female cancer survivors aged 20–49 years to ensure the reproductive concerns measured were relevant to women of reproductive age and to ensure partner support was a relevant construct across the sample. Within the Egyptian sociocultural milieu, fertility and reproductive decision-making and support often occur within marriage. Thus, married women were included to enable the study to investigate perceived spousal/partner support in the context of a relatively uniform relationship status. This exclusion also eliminated the potential for variability associated with including male survivors or single participants, whose reproductive concerns and interests, support needs, and relationship dynamics may vary and may need to be measured differently.

Postmenopausal women were excluded to create a homogeneous group of women of reproductive age in whom fertility, pregnancy planning, and reproductive decision-making were relevant. In this study, menopause was defined as physiological menopause, as opposed to treatment-induced menopause. Women with treatment-related menstrual irregularities and/or temporary amenorrhea were not excluded, unless medically indicated as non-fertile.

## Instruments

### Woman’s social and health form

This form captures essential dimensions for comprehensively understanding women’s health profiles. It includes socio-demographic data such as age, education level, residence, family structure, employment status, and income. Additionally, it explores medical history, including chronic diseases and cancer history. Finally, the form assesses reproductive history, including parity, gravida, and number of living children.

### The Korean version of reproductive concerns after cancer scale (RCAC)

The Reproductive Concerns after Cancer Scale (RCAC), originally developed by Gorman et al. (2014), was used to assess reproductive concerns among female cancer survivors^37^. In the present study, the Arabic version was prepared from the original English RCAC scale using forward and back translation procedures. The Korean validation study by Kang et al. was used only as supporting psychometric evidence and was not the source language for translation^38^. The 14-item version includes three subscales: Fertility Potential (6 items), Health Problems (5 items), and Acceptance (3 items)^38^. Each item is scored on a five-point Likert scale ranging from 1 = strongly disagree to 5 = strongly agree, with higher scores indicating greater reproductive concerns^37,38^. Previous psychometric testing of the Korean version showed strong content validity, with an item-level content validity index (I-CVI) of 1.0 and a scale-level content validity index (S-CVI) of 1.0^38^. Exploratory factor analysis supported the three-factor structure and explained 57.6% of the variance^38^. Criterion validity was examined in that previous study using the Functional Assessment of Cancer Therapy–General (FACT-G), a cancer-specific quality-of-life measure, and the Patient Health Questionnaire-9 (PHQ-9), a measure of depressive symptoms; significant correlations were reported with FACT-G (r =  − 0.36, *p* < 0.001) and PHQ-9 (r = 0.38, *p* < 0.001)^38^. The Korean version also showed good internal consistency, with Cronbach’s alpha = 0.83^38^. In the current study, the Arabic version demonstrated strong internal consistency, with Cronbach’s alpha = 0.89.

### Partner support scale

The partner support scale, adopted from Straughen et al.^39^, evaluates four dimensions of support: financial support (partner’s ability to provide financial assistance), emotional support (empathy, understanding, and meaningful conversations), affection (warmth and affection), and childcare support (involvement in childcare and shared activities). It consists of 8 items rated on a four-point Likert scale from strongly disagree 1 to strongly agree 4, with total scores ranging from 8 to 32, where higher scores indicate greater perceived support. The scale exhibited high reliability with a Cronbach’s alpha of 0.95 in the original study and 0.885 in this study.

The Partner Support Scale was selected because it specifically measures support from the intimate partner rather than general social support. This was suitable for the present study, which focused on married female cancer survivors and examined partner support in relation to reproductive concerns and psychological distress. The scale is brief, clinically feasible, and covers emotional, financial, affectional, and childcare-related support, all of which are relevant to reproductive decision-making and coping in marital relationships.

### The Arabic version of the Kessler psychological distress scale

The K10 scale, originally developed by Kessler et al.^40^ and later translated into Arabic by Easton et al.^41^, assesses the frequency of symptoms such as nervousness, hopelessness, sadness, worthlessness, and fatigue experienced over the past month. It employs a 5-point Likert scale from 1 (none of the time) to 5 (all of the time), with total scores ranging from 10 to 50, where higher scores indicate greater psychological distress. The Arabic version demonstrated high reliability with a Cronbach’s alpha of 0.88.

## Study procedures

### Tool preparation & pilot study

We translated the RCAC and Partner Support Scale into Arabic using forward and back translation. An initial translation was made by bilingual experts from English to Arabic. The Arabic versions were then back-translated into English to ensure equivalence of translation. The research team examined any discrepancies between the original and back-translated versions.

This was followed by cultural adaptation to ensure the items were clear, acceptable, and relevant in the cultural context of Egypt. The Arabic versions were reviewed for face validity, clarity, relevance, and cultural acceptability by an expert panel. Any necessary word changes were made without altering the items’ intent. The experts gave informed consent to validate the study.

A pilot study was carried out prior to the main data collection to evaluate the feasibility, clarity, cultural acceptability, and preliminary validity of the translated research instruments. Twenty female cancer survivors who fit the study criteria were recruited for the pilot. The response rate was 90%, and the average time to complete the questionnaire was 20–30 min. The items were described as clear, easy to understand, and culturally relevant. There were no significant problems with wording, order, and response options (see supplementary table).

### Data collection

The data collection period was three months (October to December 2024). Prior to the data collection, the authors sought administrative approval from the health facilities where cancer survivors received care or follow-up services. Potential participants were identified from patient databases and contacted during their regular visits to the oncology clinics or via direct communication through health care providers. Potential participants were provided with information on the study goals, data confidentiality, and the voluntary nature of their participation or withdrawal from the study without any impact on their treatment and follow-up care. Participants gave written informed consent prior to completing the questionnaire.

The questionnaire was administered in private and quiet environments, such as in special consultation rooms in the oncology clinics, to ensure confidentiality and prevent the possibility of sensitive reproductive or psychological information being disclosed to other patients, their families, or health-care providers. The questionnaire was completed by participants independently or with researcher assistance if necessary (e.g., low literacy or poor comprehension of questionnaire items). Researcher support consisted of reading the questions and response options without making any suggestions to the participants. It took around 20–30 min for each participant to complete the questionnaire.

Questionnaires did not contain any participants’ names or other direct identifiers. Participants were given a study code. Paper questionnaires were kept in a secure cabinet accessible only to the research team, and data were entered into password-protected computer files. Only the research team had access to the data. Data were analyzed and presented in aggregate, so individual participants could not be identified.

To enhance data integrity, researchers monitored for incomplete data and resolved any questions as they arose. There was no direct comparison of the responses of participants who filled in the questionnaire alone and those who received help from the researcher.

### Ethical considerations

Approval for this study was obtained from the Research Ethics Committee of the Faculty of nursing at Tanta University, Egypt, under code number (548-10-2024). The well-being and rights of all participants were rigorously safeguarded in adherence to local laws, regulations, and the ethical principles outlined in the Declaration of Helsinki. Clear and comprehensive information about the study objectives was provided to all participants. They were explicitly informed that participation was voluntary and anonymous. Participants were assured that all data collected would be kept strictly confidential and accessible only to authorized members of the research team. Before data collection began, participants provided written consent. It was emphasized that participants had the right to withdraw from the study at any time without any obligation. Regarding tools validity, the experts provided informed consent to validate the study.

### Statistical analysis

The Statistical Package for Social Sciences (SPSS), version 29, was used for data analysis. Categorical data are summarized as frequencies and percentages, while continuous variables are presented as Mean ± Standard Deviation (SD). Pearson’s correlation coefficient was employed to assess the relationships between continuous variables. A hierarchical multiple regression analysis was conducted to examine the predictors of psychological distress, with an emphasis on how fertility potential, health problems, and partner support contribute to psychological outcomes. For mediation and moderation analyses, the SPSS PROCESS macro^42^ was used. The mediating role of Partner Support in the relationship between Reproductive Concerns After Cancer and Psychological Distress was tested using PROCESS Model 4. Additionally, the moderating effect of Partner Support on the relationship between Reproductive Concerns and Psychological Distress was examined using PROCESS Model 1. Bootstrapping with 5000 samples was used to estimate the indirect effect in the mediation model and the confidence intervals for moderation effects. All statistical tests were conducted at an alpha level of 0.05 to determine significance.

The total and subscale scores of the Reproductive Concerns After Cancer Scale, Partner Support Scale, and Kessler Psychological Distress Scale were treated as continuous variables in the statistical analyses and calculated in accordance with scoring guidelines, and were described using mean, standard deviation, minimum, and maximum values. These scores were entered into Pearson correlation, hierarchical multiple regression, mediation, and moderation analyses. The main continuous scale variables were not dichotomized, categorized, or made into arbitrary cut-offs to retain statistical information and avoid reduced power through categorization. Descriptive statistics (frequencies and percentages) were used to report categorical socio-demographic and clinical variables. Questionnaires were checked for missing data prior to entry, and questionnaires with missing essential data were omitted from the final analysis. Consequently, there were 202 complete questionnaires with no missing data for the key variables. Therefore, no imputations were needed, and all analyses were performed on the 202 participants.

## Results

Table 1 included 202 participants with a varied distribution of socio-demographic characteristics. Most participants (28.2%) were aged between 25 and 30 years, 54.0% of the participants were working. Residence was almost equally distributed, with 54.0% of participants residing in rural areas and 46.0% in urban areas. Regarding the type of cancer, 46.0% of participants were diagnosed with breast cancer, followed by 21.3% with cervical cancer. In relation to parity, 34.2% of participants had given birth once while 33.2% had given birth twice. Lastly, regarding future pregnancy planning, 58.4% of participants indicated that they were planning for future pregnancies, while 41.6% were not.

**Table 1:** Socio-demographic and clinical characteristics of the study participants (*N* = 202).

Socio-demographic factors	*N*	*%*	
Age (in Years)	< 25	45	22.3
	25–(< 30)	57	28.2
	30–(< 35)	56	27.7
	35–(< 40)	26	12.9
	≥ 40	18	8.9
Employment status	Not working/ Housewife	93	46.0
	Working	109	54.0
Residence	Rural	109	54.0
	Urban	93	46.0
Family type	Extended	108	53.5
	Nuclear	94	46.5
Family income	Enough	102	50.5
	Enough & save	32	15.8
	Not enough	68	33.7
Level of education	Illiterate	44	21.8
	Basic education	68	33.7
	Secondary education	45	22.3
	University education	45	22.3
Type of cancer	Breast cancer	93	46.0
	Colon cancer	27	13.4
	Cervical cancer	43	21.3
	Lung cancer	33	16.3
	Other type	6	2.9
Duration of cancer diagnosis	< 2	90	44.6
	2–(< 5)	75	37.1
	≥ 5	37	18.3
Type of treatment	Chemotherapy	86	42.6
	Radiation therapy	46	22.8
	Surgical treatment	36	17.8
	Mixed treatment	34	16.8
Parity	Once	69	34.2
	Twice	67	33.2
	Three times	41	20.3
	> 3 times	25	12.4
Number of living children	One child	77	38.1
	Two children	67	33.2
	Three children	40	19.8
	More than 3 children	18	8.9
Future planning for pregnancy	Yes	118	58.4
	No	84	41.6

Table 2 reveals that the total score for Reproductive Concerns after Cancer ranged from 14.0 to 64.0, with a mean of 37.36 and a standard deviation of 11.79. The mean score for Partner Support was 18.87 (SD = 8.06), with scores ranging from 8.0 to 32.0. Lastly, Psychological Distress ranged from 10.0 to 50.0, with a mean of 29.08 and an SD of 9.60.

**Table 2:** Descriptive statistics of the study measures (*N* = 202).

Study measures	Minimum	Maximum	Mean	SD
Reproductive concerns after cancer	14.0	64.0	37.36	11.79
Fertility potential	6.0	30.0	14.48	8.42
Health problems	5.0	25.0	12.30	7.10
Acceptance	3.0	15.0	10.56	4.27
Partner support	8.0	32.0	18.87	8.06
Psychological distress	10.0	50.0	29.08	9.60

*SD* Standard Deviation.

Table 3 outlines the results of the Pearson correlation analysis between the study variables. Partner Support was negatively correlated with Reproductive Concerns after Cancer (r = − 0.446, *p* < 0.01), as well as with its subscales—Fertility Potential (r = − 0.422, *p* < 0.01) and Health Problems (r = − 0.413, *p* < 0.01). However, Partner Support was positively correlated with Acceptance (r = 0.289, *p* < 0.01). Psychological Distress showed a positive correlation with Reproductive Concerns after Cancer (r = 0.517, *p* < 0.01), Fertility Potential (r = 0.505, *p* < 0.01), and Health Problems (r = 0.512, *p* < 0.01), while it was negatively correlated with Acceptance (r = − 0.420, *p* < 0.01) and Partner Support (r = − 0.471, *p* < 0.01).

**Table 3:** Correlation analysis between studied measures (*N* = 202).

Study variables	(1)	(2)	(3)	(4)	(5)	(6)	
Reproductive concerns after cancer^1^	r	1					
	P						
Fertility potential^2^	r	0.978**	1				
	P	0.000					
Health problems^3^	r	0.960**	0.943**	1			
	P	0.000	0.000				
Acceptance^4^	r	− 0.765**	− 0.841**	− 0.873**	1		
	P	0.000	0.000	0.000			
Partner support^5^	r	− 0.446**	− 0.422**	− 0.413**	0.289**	1	
	P	0.000	0.000	0.000	0.000		
Psychological distress^6^	r	0.517**	0.505**	0.512**	− 0.420**	− 0.471**	1
	P	0.000	0.000	0.000	0.000	.000	

*r* = Pearson correlation.

**Correlation is significant at the 0.01 level (2-tailed).

Table 4 presents the results of a hierarchical multiple regression analysis predicting Psychological Distress. In Model 1, Health Problems emerged as a significant predictor (B = 0.570, *p* = 0.039), indicating that higher levels of health problems were associated with greater psychological distress. The other predictors, Fertility Potential (B = 0.248, *p* = 0.237) and Acceptance (B = 0.296, *p* = 0.294), did not show significant relationships with psychological distress in this model. In Model 2, after adding Partner Support as a predictor, the variance explained increased to 34.5% (R^2^ = 0.345, Adj. R^2^ = 0.331, F-change = 25.894, *p* < 0.001). Partner Support was a significant negative predictor of psychological distress (B = -0.364, *p* < 0.001), suggesting that higher levels of partner support were associated with lower psychological distress. These findings highlight the critical role of partner support in reducing psychological distress, while the impact of health problems was more pronounced in the initial model. The inclusion of partner support in the second model notably improved the model’s predictive ability, underscoring its importance as a protective factor against psychological distress.

**Table 4:** Hierarchical multiple regression analysis predicting psychological distress.

Predictors	Psychological distress						
	Unstandardized coefficients		Standardized coefficients	*t*	*P*	95% CI for difference	
	*B*	*SE*	*ß*			Lower bound	Upper bound
Model 1							
Constant	15.348	4.951		3.100	0.002	5.584	25.113
Fertility potential	0.248	0.209	0.218	1.185	0.237	− 0.165	0.661
Health problems	0.570	0.275	0.422	2.073	0.039*	0.028	1.113
Acceptance	0.296	0.281	0.132	1.052	0.294	− 0.259	0.851
Model 1							
Constant	28.217	5.439		5.188	0.000	17.491	38.942
Fertility potential	0.126	0.201	0.110	0.628	0.531	− 0.270	0.521
Health problems	0.419	0.263	0.310	1.589	0.114	− 0.101	0.938
Acceptance	0.072	0.272	0.032	0.264	0.792	− 0.464	0.607
Partner support	− 0.364	0.077	− 0.305	− 4.718	0.000**	− 0.516	0.212

*Effect (B)* Unstandardized Coefficient, *ß* standardized coefficient beta, *SE* Standard Error, *t* Student t test for linear regression.

95% CI for difference = 95% Confidence Interval for Difference.

Model 1 Summary*: R*^*2*^ = 0.271 (*Adj R*^*2*^ = 0.259), *F-*change = 27.477, *P* < 0.001.

Model 2 Summary*: R*^*2*^ = 0.345 (*Adj R*^*2*^ = 0.331), *F-*change = 25.894, *P* < 0.001.

*p < .05, **p < .01 indicate statistical significance.

Dependent variable: Psychological Distress.

**Fig. 1:**
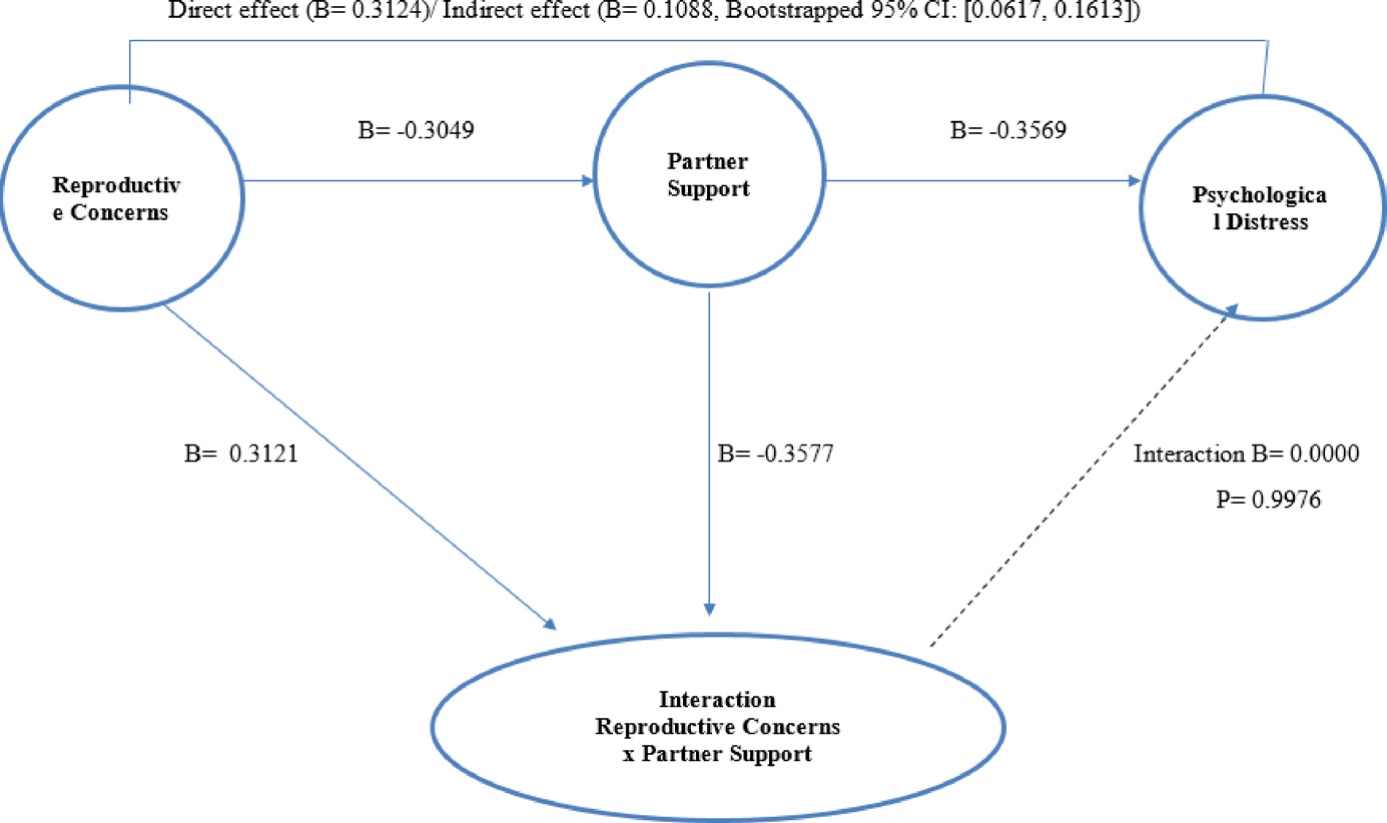
Moderation and mediation model of partner support on the relationship between reproductive concerns and psychological distress.

Table 5 and Fig. 1 display the mediation analysis results investigating the role of Partner Support (PS) as a mediator in the relationship between Reproductive Concerns After Cancer (RCAC) and Psychological Distress (PD). Model 3a shows the total effect of RCAC on PD, where RCAC significantly predicted psychological distress (B = 0.4212, *p* < 0.001), implying that higher reproductive concerns were associated with greater psychological distress. Model 3b reveals the direct effect of RCAC on PD after controlling for PS, where RCAC remained a significant predictor of psychological distress (B = 0.3124, *p* < 0.001), though the effect was reduced compared to the total effect. Lastly, model 3c presents the indirect effect of RCAC on PD through PS, which was statistically significant (B = 0.1088, 95% CI [0.0617, 0.1613]), indicating a partial mediation by partner support. The results show that partner support partly explains the relationship between reproductive concerns and psychological distress. The models collectively suggest that while reproductive concerns directly influence psychological distress, part of this effect is mediated by the level of partner support, emphasizing the importance of social support in managing psychological distress related to reproductive concerns.

**Table 5:** Mediating role of partner support in the relationship between reproductive concerns and psychological distress.

Model/ Path	Effect (B)	SE	t	p	95% CI for difference	
					Lower bound	Upper bound
Model 1 RCAC → PS	− 0.3049	0.0433	− 7.0414	0.0000	− 0.3903	− 0.2195
Model 2 PS → PD	− 0.3569	0.0767	− 4.6548	0.0000	− 0.5081	− 0.2057
Model 3a (Total Effect) RCAC → PD	0.4212	0.0493	8.5417	0.0000	0.3240	0.5184
Model 3b (Direct Effect) RCAC → PD	0.3124	0.0524	5.9569	0.0000	0.2090	0.4158
Model 3c (Indirect Effect) RCAC → PS → PD	0.1088	0.0250	–	–	0.0617	0.1613

*RCAC* Reproductive Concerns After Cancer, *PS* Partner Support, *PD* Psychological Distress, *Effect (B)* Unstandardized Coefficient, *SE* Standard Error, *t* Student t test for linear regression.

95% CI for difference = 95% Confidence Interval for Difference.

Model 1 Summary for Predicting Mediator (Outcome Variable): R^2^ = 0.1987, F (1, 200) = 49.582, *p* = 0.0000.

Model 2 Summary for Predicting Outcome: R^2^ = 0.3392, F (2, 199) = 51.0831, p = 0.0000.

Model 3 Summary for Total Effect: R^2^ = 0.2673, F (1200) = 72.9598, *p* = 0.0000.

Bootstrapped Confidence Interval for Indirect Effect: Bootstrapped 95% CI [0.0617, 0.1613] (Statistically Significant).

Table 6 and Fig. 1 illustrate the results of a moderation analysis examining whether PS moderates the relationship between RCAC and PD. The results show that the interaction term RCAC * PS (B = 0.0000, *p* = 0.998) was not statistically significant, indicating that partner support did not moderate the relationship between reproductive concerns and psychological distress. In other words, the effect of reproductive concerns on psychological distress did not vary depending on the level of partner support. Overall, while partner support plays a mediating role in reducing psychological distress (as indicated in Table 5), it does not appear to have a moderating effect on the strength of the relationship between reproductive concerns and psychological distress.

**Table 6:** Moderation analysis of partner support on the relationship between reproductive concerns and psychological distress.

Model	Effect (B)	SE	t	*p*	95% CI for difference	
					Lower bound	Upper bound
Constant	24.1645	5.0656	4.7703	< 0.001	14.1750	34.1539
RCAC	0.3121	0.1122	2.7805	0.0060	0.0907	0.5335
PS	− 0.3577	0.2746	− 1.3024	0.1943	− 0.8993	0.1839
Interaction (RCAC * PS)	0.0000	0.0069	0.0030	0.9976	− 0.0136	0.0136

*RCAC* Reproductive Concerns After Cancer, *PS* Partner Support, *PD* Psychological Distress.

Effect (B) = Unstandardized Coefficient; SE = Standard Error; *t* = Student t test for linear regression 95% CI for difference = 95% Confidence Interval for Difference.

Model Summary: R^2^ = 0.3392, F = 33.8843, p(Model) = 0.0000.

Test of Interaction Effect: R^2^ Change = 0.0000, F (Interaction) = 0.0000, p (Interaction) = 0.9976 (Not-Statistically Significant). Dependent variable: *PD* Psychological Distress.

## Discussion

Cancer survivorship often brings about a range of emotional and psychological challenges, particularly concerning reproductive health. Many survivors grapple with reproductive concerns, including fertility issues and health implications post-treatment, which can lead to significant psychological distress. This distress can manifest as anxiety, depression, and a diminished quality of life. Understanding the factors that mediate this relationship is crucial for developing effective support strategies. The current study aims to investigate the mediating role of partner support in the relationship between reproductive health concerns and psychological distress among cancer survivors.

The mean total score for reproductive concerns after cancer was 37.36 out of a possible range of 14–70. This suggests that reproductive concerns were present among the study participants; however, the score should not be interpreted as representing a formal “moderate” or “high” category because no established cut-off point was used in this study. This interpretation is based on the relative position of the mean score within the scale’s possible range, indicating a substantial burden of reproductive concerns among the study sample. This aligns with existing literature that highlights the psychological burden associated with fertility issues in cancer survivors, which can profoundly impact their quality of life and emotional well-being^10^.

The subscale scores for fertility potential and health problems reveal that these specific areas contribute significantly to overall reproductive concerns. The moderate mean scores indicate that survivors are particularly worried about their ability to conceive and the potential health implications of their cancer treatment. This is consistent with previous research that has shown that concerns about fertility can be as distressing as the cancer diagnosis itself, affecting survivors’ emotional states and overall life satisfaction^10^. Moreover, the mean score for partner support indicates that while many survivors perceive some level of support, there is still significant room for improvement. The relationship between partner support and reproductive concerns denotes that higher levels of perceived support are associated with lower levels of anxiety regarding reproductive issues. This finding emphasizes the importance of social support in mitigating the emotional distress associated with cancer survivorship. Research consistently shows that strong partner support can enhance coping mechanisms and reduce feelings of isolation among cancer survivors^43^.

Furthermore, the mean score for psychological distress indicates that many survivors experience moderate to high levels of emotional distress. This finding is consistent with other studies that report elevated rates of anxiety and depression among cancer survivors^43,46^. The relationship between psychological distress and reproductive concerns implies that unresolved reproductive issues contribute to overall emotional turmoil.

The current study findings indicate a significant negative correlation between partner support and reproductive concerns after cancer, denoting that higher levels of perceived partner support are associated with lower levels of reproductive anxiety. This aligns with existing literature that emphasizes the role of social support in mitigating distress and enhancing coping mechanisms among cancer survivors^44^. The analysis further reveals that partner support is negatively correlated with both fertility potential and health problems, reinforcing the idea that supportive partners can help alleviate specific concerns related to fertility and health outcomes. Conversely, partner support shows a positive correlation with acceptance, indicating that those who feel more supported by their partners are more likely to accept their reproductive situation. This finding signifies the importance of relational dynamics in cancer survivorship, where emotional and practical support from partners can foster a greater sense of acceptance and resilience^45^.

Psychological distress is positively correlated with reproductive concerns, fertility potential, and health problems, indicating that as reproductive concerns increase, so does the level of psychological distress experience by survivors. This relationship highlights the emotional toll that unresolved reproductive issues can have on mental health, denoting that addressing these concerns is crucial for improving overall well-being^46^. Additionally, the negative correlation between psychological distress and acceptance indicates that higher levels of acceptance are associated with lower levels of distress. This finding supports the notion that fostering acceptance can serve as a protective factor against psychological distress in cancer survivors^47^.

The regression analysis results of the current study indicate that health problems significantly contribute to psychological distress, indicating that as individuals experience more health-related issues, their psychological well-being declines. This finding is consistent with existing literature that highlights the emotional burden associated with health complications in cancer survivors, where physical health challenges can exacerbate feelings of anxiety and depression^48^. In contrast, the predictors of fertility potential and acceptance did not demonstrate significant relationships with psychological distress in the first model. This may imply that while these factors are relevant to the overall experience of cancer survivorship, their direct impact on psychological distress may be less pronounced compared to health problems. Previous studies have shown that acceptance can play a role in coping with cancer, but its effect may not be as immediate or measurable in terms of psychological distress as health problems^47^.

The introduction of partner support in Model 2 significantly enhanced the model’s predictive ability, indicating that social support is crucial in mitigating psychological distress. The negative association between partner support and psychological distress implies that higher levels of support from partners are linked to lower levels of distress. This finding aligns with research emphasizing the protective effects of social support in cancer survivorship, where emotional and practical support from partners can buffer against the psychological impacts of the disease^44^.

The current mediation analysis indicates that reproductive concerns are positively associated with psychological distress, denoting that as individuals experience heightened worries regarding their reproductive health post-cancer, their psychological well-being is adversely affected. This aligns with existing literature that emphasizes the emotional toll of reproductive concerns on cancer survivors, where anxiety about fertility and reproductive health can lead to increased levels of distress^49^. The analysis further reveals that partner support is a mediator in this relationship. Specifically, the presence of supportive partners appears to mitigate the negative impact of reproductive concerns on psychological distress. This finding underscores the importance of social support systems, particularly from intimate partners, in enhancing mental health outcomes for individuals facing reproductive challenges after cancer. Research has consistently shown that strong partner support can buffer against psychological distress, providing emotional and practical assistance that helps individuals cope with their concerns^50^.

Moreover, the results indicate that while reproductive concerns directly influence psychological distress, a portion of this effect is explained through the mediating role of partner support. This partial mediation denotes that interventions aimed at enhancing partner support could be beneficial in reducing psychological distress among cancer survivors dealing with reproductive concerns^51^.

The moderation analysis reveals that the interaction between reproductive concerns and partner support is not statistically significant, indicating that partner support does not moderate the relationship between these variables. This indicates that the impact of reproductive concerns on psychological distress remains consistent, regardless of the level of partner support available to the individual. The finding that partner support does not moderate the relationship between reproductive concerns and psychological distress is noteworthy. It implies that while partner support may be beneficial in reducing psychological distress, it does not change the strength or direction of the relationship between reproductive concerns and distress. This could denote that the psychological impact of reproductive concerns is a more direct experience, potentially overwhelming any buffering effect that partner support might provide. Previous research has indicated that reproductive concerns can be deeply distressing for cancer survivors, often leading to anxiety and emotional turmoil that may not be easily alleviated by external support systems^52^.

Furthermore, the results highlight the complexity of psychological distress in the context of cancer survivorship. While partner support has been shown to play a mediating role in reducing distress, as indicated in earlier analyses, its lack of moderating effect indicates that the relationship between reproductive concerns and psychological distress operates independently of the support received from partners. This finding emphasizes the need for targeted interventions that address reproductive concerns directly rather than solely relying on partner support as a means of alleviating distress.

## Strengths and limitations

The methods utilized in this study demonstrate rigorous attention to detail and a commitment to methodological robustness. Employing a cross-sectional descriptive design and adhering to the STROBE checklist ensures methodological clarity and transparency. Conducting the study in the specialized Oncology Departments of Tanta University Hospital adds credibility due to the institution’s established reputation for comprehensive cancer care and research. The inclusion and exclusion criteria are meticulously designed, focusing on a specific demographic to ensure homogeneity and the validity of results. The sample size calculation, supported by G*Power software, reflects a well-planned approach to account for multiple predictors, dropout rates, and statistical power, enhancing the study’s reliability. Furthermore, the use of validated and culturally adapted tools like the Arabic versions of the RCAC, Partner Support Scale, and Kessler Psychological Distress Scale underscores the cultural sensitivity and psychometric rigor of the instruments.

Despite the study’s strengths, certain limitations warrant consideration. The cross-sectional design precludes causal inferences, restricting the ability to establish temporal relationships between variables. The reliance on self-reported measures may introduce response bias, as participants could overreport or underreport their experiences. Future research could strengthen methodological rigor by incorporating complementary approaches such as clinician-administered assessments, partner-reported measures, or objective clinical indicators where applicable. In addition, longitudinal designs would allow for the examination of temporal and causal relationships, while mixed methods approach, including qualitative interviews, could provide deeper insights into survivors lived experiences and contextual factors influencing reproductive concerns and psychological distress. Although the translation and back-translation process aimed to ensure cultural appropriateness, nuances specific to Arabic-speaking populations might still be overlooked. The recruitment of participants from a single geographical location and healthcare setting may limit the generalizability of the findings to broader populations. Additionally, the study’s focus on married, reproductive-age women excludes other demographics of cancer survivors who may face distinct challenges, potentially narrowing the scope and applicability of the results.

The study was limited to married female cancer survivors of reproductive age. Therefore, the findings may not be generalizable to male cancer survivors, unmarried women, divorced or widowed survivors, or individuals in non-marital relationships. Reproductive concerns and partner support may differ across these groups, and future studies should examine these populations using designs and instruments appropriate to their relational and reproductive contexts.

Another limitation is that participants were allowed to complete the questionnaire either independently or with assistance from a trained researcher. Although assistance was limited to reading or clarifying the wording of items, the study did not examine whether responses differed systematically between independently completed and staff-assisted questionnaires. Therefore, potential response bias related to researcher-assisted completion cannot be fully excluded.

The exclusion of women with age-related menopause and women medically unable to conceive may limit the generalizability of the findings. These groups may still experience reproductive health concerns, partner-related challenges, and psychological distress, but their reproductive experiences may differ from those of women with potential fertility. Future studies should examine these groups separately using designs and measures appropriate to their reproductive status.

## Conclusions

The study highlights the significant association between reproductive concerns and psychological distress among female cancer survivors. Partner support partially mediated this relationship, suggesting that reproductive concerns may be linked to psychological distress partly through reduced perceived partner support. However, the moderation analysis was not significant, indicating that partner support did not change the strength of the relationship between reproductive concerns and psychological distress. These findings support the need for targeted psychosocial interventions that directly address reproductive concerns while also strengthening partner support as part of survivorship care.

## Implications

Nursing education should prioritize training programs that address the psychosocial and reproductive concerns of cancer survivors, integrating knowledge about fertility preservation options and psychological support into curricula. This can equip nursing students and professionals with the skills to provide holistic, patient-centered care. Effective communication techniques, particularly for discussing sensitive issues such as reproductive health, should be emphasized to foster trust and patient engagement. In practice, nurses should adopt proactive approaches to identifying and addressing reproductive concerns during routine care, collaborating with patients and their partners to develop personalized care plans. Furthermore, involving partners in education and counseling sessions can strengthen support systems, enhance patients’ psychological well-being and improve adherence to care recommendations.

Future research should focus on exploring the mediating role of partner support in mitigating reproductive and psychological concerns among cancer survivors, as well as identifying the specific factors that influence the effectiveness of such support. Longitudinal studies are essential to evaluate the long-term psychological outcomes of integrating partner support and fertility preservation counseling into cancer care. Additionally, research should be expanded to include diverse cultural and demographic populations, ensuring that findings can inform culturally sensitive nursing interventions. Investigating innovative strategies, such as digital tools or community-based programs, may further enhance nursing practices and improve patient outcomes in this population.

## Supplementary Information

Supplementary Information.

## Data Availability

The data that support the findings of this study are available from the corresponding author upon reasonable request.
