# Supplementary material for: The mediating role of partner support in the relationship between reproductive health concerns and psychological distress among cancer survivors
Source: Sci Rep. 2026 May 25;16:16142. doi: 10.1038/s41598-026-53124-z (PMC13201610; doi:10.1038/s41598-026-53124-z)
Supplement: Supplementary file 1 — Supplementary Information. [file 41598_2026_53124_MOESM1_ESM.docx]

**Supplementary Table. Pilot Study Findings**

| **Pilot study item** | **Result** |
| --- | --- |
| Pilot sample | 20 female cancer survivors |
| Response rate | 90% |
| Completion time | 10–15 minutes |
| Item clarity | Items were clear and understandable |
| Cultural suitability | No culturally inappropriate wording was reported |
| Reported difficulties | No major difficulties with wording, order, or response options |
| RCAC Cronbach’s alpha | 0.89 |
| Partner Support Scale Cronbach’s alpha | 0.885 |
| Arabic Kessler Psychological Distress Scale Cronbach’s alpha | 0.88 |
| Changes after pilot | No major revisions required |
